# Diversity in Representations and Voices of Terminally Ill People in End-of-Life Documentaries

**DOI:** 10.1177/08258597211013961

**Published:** 2021-05-03

**Authors:** Outi J. Hakola

**Affiliations:** 1 Area and Cultural Studies, University of Helsinki, Helsinki, Finland

**Keywords:** documentary film, equality & diversity, hospice care, palliative care, race and ethnicity, representation, terminally Ill, voice

## Abstract

**Background:** The 21st century has seen a proliferation of end-of-life documentary films and television documentaries that contribute to building a public image of hospice and palliative care. The way in which terminally ill patients are represented in these documentaries creates impressions of who is welcomed to receive end-of-life care. These documentary representations have not been previously mapped. **Methods:** Using quantitative content analysis, I analyzed 35 contemporary Western documentaries and studied their diversity in the representations. I focused on terminally ill patients who are given time and space in the narration to voice their views about the end-of-life process. I paid attention to such elements as gender, race and ethnicity, age, class, religion and sexuality. **Results:** The documentaries welcomed the representations and voices of terminally ill people. Class, religion and sexuality often had a marginal role in narration. The gender diversity of the representations was quite balanced. Regarding age, the documentaries preferred stories about working age patients for dramatic purposes, yet all age groups were represented. However, the documentaries had an identifiable racial and ethnic bias. With a few exceptions, terminally ill who had a personal voice in the narrations were white. In comparison, racial and ethnic minorities were either absent from most of the documentaries, or their role was limited to illustrations of the general story. **Conclusions:** End-of-life documentaries provide identifiable access to the patients’ experiences and as such they provide emotionally and personally engaging knowledge about hospice and palliative care. While these representations are people-oriented, they include racial disparities and they focus mostly on the experiences of white terminally ill patients. This bias reinforces the misleading image of hospice and palliative care as a racialized healthcare service.

## Introduction

The 21st century has seen a proliferation of documentary films and television documentaries that focus on stories about the end of life.^1^ They build a public image of hospice and palliative care as interdisciplinary approach to the medical, psychological, spiritual, and emotional care of the seriously ill. For example, they have recognizable educational potential to teach about the emotional aspects of the end of life for healthcare students.^2^ They have also provided visibility and empowerment for dying people and given publicity to palliative care in the process.^3-6^ Overall, the documentary projects have raised the visibility of end-of-life care.

While people are increasingly familiar with hospice and palliative care, misconceptions remain common.^7-9^ Particularly, challenges related to equality and racial disparities are notable. In Western countries (majority of Europe, North America and Australasia), white people are more familiar with the subject while misconceptions are more typical in racial and ethnic minority groups.^10-19^ Disparities stem from complex issues, and among them are cultural barriers, including cultural attitudes, educational aspects and exclusive marketing materials.^20,21^ Consequently, several studies have recommended inclusive representations of the experiences of minority groups.^9,22,23^ Yet audiovisual representations are quite one-dimensional. In Hollywood films, white (and often young and beautiful) characters can have terminal illnesses whereas the natural deaths of other races are rather invisible.^24,25^ Similarly, both hospice commercials^26^ and informational videos^23,27^ prefer representing white people.

The representations build a public image of end-of-life care, and the unequal tendencies of these representations create hierarchical power relations within society. Aaron labels these practices where media narratives take part in the cultural politics of death as “mortal economies.”^24^ These practices have a long tradition in the philosophy of death. For example, such theories as the privatization of death by Ariés^28^ have been criticized for being based on the lives and deaths of white and wealthy men.^24,29^ In this study, I turn the focus to end-of-life documentaries and analyze the diversity of representations of the terminally ill.

## Materials and Methods

### Documentaries

My objective was to analyze how demographically inclusive public image the documents’ casting choices of terminally ill patients create of end-of-life care. For the purposes of this study, I analyzed 35 documentary films and television documentaries released between the years 2000 and 2019 (see Table 1). The documentaries originate from high-income Western countries, which share at least somewhat similar demography, and cultural, social and medical practices related to the end of life. Four documentaries are from Australia and New Zealand, 14 from Europe (Belgium, Denmark, Finland, Germany, Italy, Spain, Sweden, the United Kingdom, and Switzerland), and 17 from North America (Canada and the United States). Most of the documentary filmmakers were men (27 men and 19 women), and most of them were white (43 out of 46).

**Table 1.** The Analyzed Documentaries.

Documentary	Year	Country	Director(s)	Production company
A good death	2010	Australia	Deborah Masters, Matthew Carney	ABC Australia
Bearing witness: Robert Coley-Donohue	2003	Canada	Dan Curtis	National Film Board of Canada
Before we go	2014	Belgium	Jorge León	Center de l’Audiovisuel à Bruxelles; Dérives Productions; Les Films du Fleuve; New Impact; Unité Documentaire RTBF-Liège
Being mortal	2015	USA	Thomas Jennings, Nisha Pahuja	PBS / Frontline
Bouton	2011	Switzerland	Res Balzli	Balzli & Fahrer Filmproduktion
Consider the conversation 2: stories about cure, relief, and comfort	2014	USA	Mike Bernhagen, Terry Kaldhusdal	Burning Hay Wagon Productions
Consider the conversation: a documentary on a taboo subject	2011	USA	Mike Bernhagen, Terry Kaldhusdal	Burning Hay Wagon Productions
Dying at grace	2003	Canada	Allan King	Allan King Associates
En god død	2013	Denmark	Estephan Wagner	Final Cut for Real ApS; New Danish Screen
End game	2018	USA	Rob Epstein, Jeffrey Friedman	Netflix
Except for 6	2008	USA	Matthew Burnell	Visioneer Films
Facing death	2010	USA	Miri Navasky, Karen O’Connor	PBS / Frontline
Griefwalker	2008	Canada	Tim Wilson	National Film Board of Canada
Helen’s story	2016	New Zealand	Paul Davidson, Barbara Gibb	Bytesize Productions
Hirschhausen im hospiz	2019	Germany	Stefan Otter, Krischan Dietmaier, Theresa Moebus	ARD/Das Erste
I remember when I die	2015	Sweden	Maria Bäck	Garagefilm International
Inside the human body: first to last	2011	UK	Annabel Gillings	BBC
ISLAND	2017	UK	Steven Eastwood	Hakawati
Kuoleman kasvot	2003	Finland	Kiti Luostarinen	Kiti Luostarinen Productions
Les pal·liatives	2018	Spain	Oriol Gispert, Marta Valls	Lupa Producciones, La; Televisión de Cataluña
Letzte saison—wenn es zeit ist zu sterben	2011	Germany	Sigrid Faltin	ARD/Das Erste
Letzte tage, gute tage—palliativ-versorgung in deutschland	2015	Germany	Annette Hoth	ZDF
Life before death	2012	Australia	Mike Hill	Moonshine Movies
Living while dying	2017	USA	Cathy Zheutlin	Peace Films
Living your dying	2003	USA	Robert Pennybacker	Lotus Film Productions
Love in our own time	2011	Australia	Tom Murray, Madeleine Hetherton	Tarpaulin Productions
Mortal lessons	2008	USA	David Liban	University of Colorado Denver
Prison terminal: the last days of private jack hall	2013	USA	Edgar Barens	HBO
7 songs for a long life	2015	UK	Amy Hardie	SDI Productions
Solace: wisdom of the dying documentary	2018	USA	Camille Adair	Sacredigm Alliances
The end	2004	USA	Kirby Dick	HBO
The light inside	2015	Canada	Neil Hicks	Hospice Peterborough
The LuLu sessions	2011	USA	S. Casper Wong	OO Media
The perfect circle	2014	Italy	Claudia Tosi	Movimenta
Tuntemattoman edessä	2000	Finland	Tiina Merikanto	Yleisradio / Silminnäkijä

The data do not include all produced end-of-life documentaries within Western countries. First, due to language limitations the data includes documentaries that were or were subtitled in the following languages: Danish, English, Finnish, German, Spanish, and Swedish. Second, I used films that have inspired public discussion. I made the selection based on hospice and palliative care foundations’ online recommendations, articles in major newspapers, and screening lists from film festivals. From the potential material, I excluded documentaries about children’s palliative care because these documentaries focused on parents’ voices, highlighting a different set of questions. Third, I excluded end-of-life documentary television series, such as *Time of Death* (2013, U.S.), where each episode narrated the story of a different dying person. The series format utilizes a different logic for diversity in character casting than documentaries or episodes that independently approach the issue.

In the documentaries, the selection of represented dying people is the central (and political) narrative choice because, according to film theories, the characters serve as the viewer’s entry into the cinematic narratives.^30^ Instead of using omnipotent narration, the contemporary documentaries prefer inviting their filmed subjects (characters) to be equal partners in the conversation in order to create interaction and understanding of lived experiences.^31^ Similarly, in end-of-life documentaries, the terminally ill patients affectively engage viewers with their personal and emotional experiences in addition to the institutional voices of palliative care professionals.

Voice is a central concept in documentary studies and refers to the space and time that a certain perspective, such as each character’s perspective, has in the narration. Voice is also more than dialogue; it is a combination of several cinematic forms of expression, such as editing decisions, shot length, framing, music, and represented spaces and people.^32^ This concept allows differentiation between those with a personal voice and those who serve as a background to the narratives. For example, while many American Spanish-speaking hospice videos represented Latinx patients, they used narrators to explain the care and left the patients to serve as background images.^23^ Thus, the Latinx patients lacked an individual voice. As voice provides the potential for viewer engagement, in this study I focus on those groups that have had their voices heard and those groups that have been marginalized as participants in public conversations about end-of-life experiences.

### Content Analysis

I used quantitative content analysis to study the cinematic voice of the terminally ill. First, I identified all of the terminally ill with a personal voice. In practice, I listed all of the introduced dying people who had a possibility to communicate their own experiences, identities and stories either through dialogue or through foreground images. I excluded those used as illustrations (background images) or mentioned in passing by other characters.

Second, I recorded the gender, race/ethnicity, age and other potentially available personal information such as class, religion or sexuality of those with a personal voice. The documentaries did not always provide all of this information. Furthermore, I recorded gender and race based on visual representation, not by their self-identification. Consequently, the potential misinterpretations may have influenced the analysis and this method leaves non-binary gender identities and multiracial identities hidden. I was able to consider these aspects only if the characters self-identified the various layers of their identities.

Third, I calculated the time dedicated to the cinematic voice of each listed character. I included scenes where they were talking, active participants in the scene, or the visual focus of the camera without anyone else narrating their stories to the viewer. Allocated time served as a comparative tool between various documentaries.

In the fourth and last phase, I used the results from the previous phases to find relevant examples of terminally ill voices. These examples deepen understanding of the uses and functions of terminally ill voices in the end-of-life documentaries.

## Results

In total, 135 palliative care patients had a recognizable cinematic voice in the documentaries. The combined length of the documentaries was 39 hours and 10 minutes, of which the dying had 19 hours and 52 minutes (51%). The research data is summarized in 2 supplemental tables, which describe the quantitative info of documentaries at the level of films (Supplemental Table 1) and at the level of characters in the films (Supplemental Table 2).

The documentaries used various narrative structures. Each documentary had a policy with regard to how it framed the different voices (the narrative perspectives of documentaries are listed in the Supplemental Table 1). Most of them (16) took an institutional perspective where hospice and palliative care professionals and institutions define and discuss the end of life. These documentaries used several terminally ill people (on average 4.75 per documentary) to illustrate different issues raised in the narration. They each had limited time and space for their voice (3:28 minutes each on average, totaling 29% of the narration).

Another set of documentaries (14) had a patient-oriented approach, witnessing the experiences of the terminally ill and letting the viewers draw their own conclusions without an institutional voice explaining the events. These documentaries focused on 1 or a small group of the terminally ill (on average 3 per documentary), who were given plenty of time and space (18:55 minutes each on average, totaling 71% of the narration).

The third frame was that of the filmmakers (5). These documentaries used first-person narration and brought the personal growth of the filmmaker to the fore. The terminally ill had an interactive and personal impact on their journeys, and on average 3.4 terminally ill had a voice per documentary. On average, they received 7 minutes and 56 seconds to voice their views (totaling 40% of the narration).

### Race and Ethnicity

In all of the documentaries, race and ethnicity raised the most questions in relation to the diversity of representations. Out of 35 documentaries, 26 gave voice only to the white patients. Nine documentaries had more diversity: a New Zealand documentary focused on a half-Maori, half-white woman; an Australian documentary took a global aspect and narrated palliative care stories from Africa, Asia, and North America; and 7 American documentaries introduced various patients, including black, indigenous and people of color. Conversely, all European and Canadian documentaries only told stories about white terminally ill patients.

In total, the documentaries introduced 107 white patients and 28 people with a minority background: 14 black patients, 9 Asian-origin patients, 3 Latinx patients and 2 indigenous patients. Out of the total duration of the documentaries, white terminally ill patients spoke 46% of the time, and black, indigenous and people of color 5% of the time. At the individual level, whereas white patients had on average 10 minutes to voice their views, patients from racial and ethnic minority groups had only 4 minutes.

The breakdown of this general view shows that most white patients (58% of white characters) had more than 5 minutes to voice their views, and 22% of them (23 people) were given more than 15 minutes of narrative time. Five documentaries focused on 1 white person’s dying process. In comparison, most black, indigenous and people of color received less than 5 minutes of focus (79%), and only 1 (half-white, half-Maori) character received more than 15 minutes of narrative time and a whole documentary dedicated to their experiences.

Most black, indigenous and people of color appeared in the institution-oriented documentaries (25 characters), where they provided exemplary perspectives on the main narrative. For example, in *Living Your Dying* and in *Solace: Wisdom of the Dying* the terminally ill spoke about their spiritual experiences. A native Hawaiian woman talked about finding strength in her inner self.^33^ Two Latino men contemplated the energy they would be leaving behind. An Asian-American woman addressed encountering relationships and life with an open heart. In addition, a black woman discussed how her experience of mortality inspired a personal spiritual journey.^34^ These documentaries used their voices to encourage personal growth and the acceptance of mortality.

The medical-oriented institutional stories often reduced the voice of black, indigenous and people of color to reactive comments. In reactive comments an authority figure introduces a theme, which is reinforced through a personal experience of the patient. For example, In *End Game*, a doctor explains that people have often difficulties to accept hospice referrals because they have misconceptions about hospice. After this, the camera cuts to the Doctor’s meeting with a patient, where he explains what a hospice is, and the patient and their family admit that they might not be ready for hospice because it means saying goodbye.^35^ Narratively, the patient’s role is to react to the pre-selected theme. These reactions tend to be short comments, instead of stories of personal experiences.

Reactive comments continue racial tendencies of narration. For example, *Facing Death*, which narrated palliative care in the ICU context, introduced 4 black and 3 white terminally ill patients. The black patients were physically almost unable to express themselves and they received less than 2 minutes to voice their views. In comparison, only 1 white character was in a similar condition, and the other 2 white characters were able to discuss their situations for 2:41 minutes and 5:29 minutes, respectively.^36^ These 2 characters discussed more than their medical condition, also voicing their fears, hopes and emotions.

While white characters occasionally provided reactionary comments, in the patient-oriented and filmmaker-oriented documentaries they got to describe their emotional and personal journeys toward death. They discussed their changed priorities, desire to make amends, struggles with losing their independence, unwillingness to leave their families, or fears that they were missing out on life. Only a few documentaries provided similar options for people with racial minority backgrounds. *Helen’s Story* introduced a young half-white, half-Maori teacher with terminal cancer. At the beginning of the film, she felt that she could not afford to give up because of her responsibilities and plans for the future. When she grew tired, she became increasingly emotional and started to hope for a peaceful death and aimed to accept her fate.^37^

*The End* provided another exception whereby people from marginalized groups narrated emotional details. The interactive documentary had 5 segments where patients and their families filmed their own stories in an intimate manner. The grieving parents narrated most of a Latinx child’s story, but a black man who opened the documentary became a vocal point for the film. His segment (12:40 min) introduced an angry man who lashed out at his family because he had difficulties in adjusting to his reduced independence. He was hoping for a miracle that never came, and only after admitting being scared did he start to communicate better with his family.^38^ His story is similar to those of the white terminally ill who got to voice a variety of emotions, including negative ones.

### Gender

The documentaries gave voice to 62 terminally ill men (46%) and 73 terminally ill women (54%). On average, both men and women received about 9 minutes to speak. While the documentaries represent more women patients, proportionally more women had less than 2 minutes (33% of women compared to 24% of men), and proportionally more men were given more than 15 minutes (19% of men compared to 16% of women). Based on these figures, it appears that more women were limited to reactive commentary in the documentaries, but both men and women got to play the role of the main character (in total, 12 men and 12 women got more than 15 minutes of narration time). Thus, the documentaries created a rather equal overall view, and gender did not appear to influence the ways in which the characters could voice their experiences and emotions.

However, there was some cause for intersectional concern. There were almost equal numbers of white women (55, 41% of all characters) and white men (52, 39% of all characters) and they received a rather similar time slot in the documentaries. In comparison, the documentaries represented 10 men (7% of all characters) and 18 women (13% of all characters) from racial minorities, and the number of women of color (10) in reactionary roles (allocated less than 2 minutes) explains the difference in proportional times between genders. In racial minority groups, the volume of people was small, but it suggests that the natural death of men of color is often hidden from the public discussion.

### Age, Class, Sexuality, and Religion

The age of the terminally ill was often, but not always mentioned in the documentaries. Due to the selection process, the material included only a few underage children (4), and their voice was rather limited (on average 1:13 minutes). Most of the terminally ill (79, 59%) were of working age (ca. 18-65 years old), and their voice was well represented in the documentaries (9:25 minutes). The 52 (39%) elderly patients (ca. over 65 years old) were all well represented (8:32 minutes). There were no major differences when comparing gender and race to the age representations.

In several cases, the documentaries did not refer to the class, sexuality or religion of the terminally ill. The visual imagery represented symbols familiar from Christianity, Judaism, Islam, Buddhism, and indigenous religions, but religion was rarely the key point of narration. The documentaries did not discuss the sexuality of the terminally ill. It was only present through family relationships. Similarly, class was another element that was absent. Occasionally, people discussed their professions, and those that were mentioned tended to be either middle-class or working-class professions. However, none of the documentaries focused on the social status or identities of the terminally ill. By and large, the documentaries tended to treat approaching death as a universal human trait, leaving race representations to stand out from the narrative solutions.

## Discussion

In this analysis of 35 end-of-life documentaries released from 2000-2019, the voice of the terminally ill themselves was a significant part of the narration. It was particularly strong in the patient-oriented documentaries, the main perspective of which was to witness and communicate the experiences of the dying. Still, even those documentaries with an institutional approach involved various patient voices and these institutional documentaries had the most racially diverse approach to the end-of-life representations.

Race/ethnicity and age appeared to be the only significant factors influencing the representations of the voices. While life expectancy in Western countries is close to or over 80 years,^39^ most of the characters were of working age (ca. under 65 years old). Most likely, this is a dramatic narrative choice because it is more tragic and emotional to witness the experiences of a person who is dying prematurely.

Race was the most significant element affecting diverse representations of the terminally ill. In the end-of-life documentaries, the voices of white patients were openly available. They had space and time to talk about their experiences and to paint a complex image of the dying process. With a few exceptions, the patients from other racial groups were either invisible, in the background, or had roles that illustrated general arguments in the documentaries.

From the narrative perspective, this racial tendency has significant consequences. Narratology recognizes flat and round characters, where flat characters have a limited function in the story and round characters have depth and complex personalities that can develop throughout the story.^40^ According to Plantiga, documentaries create round characters by filming them in various contexts and by giving them time and space in the narration.^41^ In turn, round characters inspire sympathy in the viewers and invite viewers to engage with their experiences.^42^ At the same time, when the end-of-life documentaries tend to exclude racial and ethnic minority groups, they offer white people’s experiences as assumed and normalized experiences of dying. This reinforces the public image of hospice and palliative care as a racialized healthcare service.

### Limitations and Policy Implications

This study has some limitations that influence the results. First, the study builds on a selection of documentaries, and hence those that I am not aware of, did not have access to, or did not have language skills for analysis might have changed the overall view. Second, content analysis limits the analysis to spoken identities, whereas other approaches might give access to study the unspoken elements of diversity. Third, the study aims to critique the general view that the end-of-life documentaries convey, not any individual documentary in terms of unique production contexts, access to patients, and narrative frames.

The hospice and palliative care’s history as a grassroots movement would be reconfirmed with an inclusive image of those who receive this care. The hospice and palliative care providers would benefit from not only paying attention to superficial diversity of public images, but from increasing the use of stories and voices of people with various backgrounds. In the future, increasingly intersectional research could help us to recognize diversity more deeply.

## Conclusion

This study demonstrated racial biases in documentaries focusing on end-of-life care that are similar to biases found in other media representations. While white patients often appeared as central characters in the narratives presented, black, indigenous and people of color patients were more frequently marginalized and given much less opportunity to reflect on their experiences with terminal illness. As we build public awareness and continue to represent seriously ill patients across many forms of visual media, I hope that this study can serve as a foundation of knowledge for the current state of representation and help characterize the need for more inclusive representation of a diverse group of people facing serious illness.

## Supplemental Material

Supplemental Material, sj-docx-1-pal-10.1177_08258597211013961 - Diversity in Representations and Voices of Terminally Ill People in End-of-Life Documentaries Click here for additional data file. Supplemental Material, sj-docx-1-pal-10.1177_08258597211013961 for Diversity in Representations and Voices of Terminally Ill People in End-of-Life Documentaries by Outi J. Hakola in Journal of Palliative Care
