# Supplementary material for: Diversity in Representations and Voices of Terminally Ill People in End-of-Life Documentaries
Source: J Palliat Care. 2021 May 3;37(2):190–6. doi: 10.1177/08258597211013961 (PMC9109586; doi:10.1177/08258597211013961)
Supplement: Supplemental Material, sj-docx-1-pal-10.1177_08258597211013961 - Diversity in Representations and Voices of Terminally Ill People in End-of-Life Documentaries [file sj-docx-1-pal-10.1177_08258597211013961.docx]

**Appendices**

The research data is summarized in two supplemental tables, which describe the quantitative info of documentaries at the level of films (supplemental table 1) and at the level of characters in the films (supplemental table 2).

Supplemental table 1: Narrative perspectives of documentaries, gender and race of the patients at the level of the films, length of the documentaries and narrative time (voice) that was dedicated to terminally ill patients in each documentary.

| **Documentary** | **Narrative perspective** | **Patients** | **Women** | **Men** | **White** | **People of color** | **Total lenght** | **Patient time** | **% patient time** |
| --- | --- | --- | --- | --- | --- | --- | --- | --- | --- |
| A Good Death | Institution | 4 | 2 | 2 | 4 | 0 | 0:45:04 | 0:16:59 | 37,68 |
| Bearing Witness: Robert Coley-Donohue | Patient | 1 | 0 | 1 | 1 | 0 | 1:30:51 | 1:05:48 | 72,43 |
| Before We Go | Patient | 3 | 1 | 2 | 3 | 0 | 1:22:21 | 0:55:48 | 67,76 |
| Being Mortal | Institution | 3 | 1 | 2 | 3 | 0 | 0:54:01 | 0:17:33 | 32,49 |
| Bouton | Patient | 1 | 1 | 0 | 1 | 0 | 1:18:14 | 1:05:36 | 83,85 |
| Consider the Conversation 2: Stories about Cure, Relief, and Comfort | Institution | 3 | 1 | 2 | 3 | 0 | 1:00:24 | 0:17:54 | 29,64 |
| Consider the Conversation: A Documentary on a Taboo Subject | Institution | 10 | 4 | 6 | 8 | 2 | 1:26:00 | 0:13:06 | 15,23 |
| Dying at Grace | Patient | 5 | 3 | 2 | 5 | 0 | 2:27:55 | 2:04:49 | 84,38 |
| En god død | Patient | 4 | 4 | 0 | 4 | 0 | 0:58:55 | 0:55:06 | 93,52 |
| End Game | Institution | 5 | 4 | 1 | 3 | 2 | 0:40:15 | 0:21:05 | 52,38 |
| Except for Six | Institution | 3 | 2 | 1 | 2 | 1 | 0:54:07 | 0:15:03 | 27,81 |
| Facing Death | Institution | 7 | 3 | 4 | 3 | 4 | 0:53:40 | 0:12:31 | 23,32 |
| Griefwalker | Filmmaker | 3 | 3 | 0 | 3 | 0 | 1:10:00 | 0:12:09 | 17,36 |
| Helen's Story | Patient | 1 | 1 | 0 | 0 | 1 | 1:11:44 | 0:31:19 | 43,66 |
| Hirschhausen im Hospiz | Institution | 4 | 3 | 1 | 4 | 0 | 0:44:00 | 0:06:01 | 13,67 |
| I Remember When I Die | Filmmaker | 5 | 3 | 2 | 5 | 0 | 1:24:16 | 0:36:02 | 42,76 |
| Inside the Human Body: First toLast | Institution | 1 | 0 | 1 | 1 | 0 | 0:59:08 | 0:03:23 | 5,72 |
| Island | Patient | 4 | 1 | 3 | 4 | 0 | 1:29:21 | 0:59:19 | 66,39 |
| Kuoleman kasvot | Filmmaker | 4 | 1 | 3 | 4 | 0 | 0:53:00 | 0:21:38 | 40,82 |
| Les Pal·liatives | Institution | 5 | 2 | 3 | 5 | 0 | 1:02:18 | 0:24:45 | 39,73 |
| Letzte Saison - wenn es Zeit ist zu sterben | Patient | 3 | 1 | 2 | 3 | 0 | 1:28:56 | 1:08:07 | 76,59 |
| Letzte Tage, gute Tage - Palliativ-Versorgung in Deutschland | Institution | 2 | 0 | 2 | 2 | 0 | 0:28:38 | 0:01:34 | 5,47 |
| Life before Death | Institution | 14 | 10 | 4 | 4 | 10 | 1:21:02 | 0:25:37 | 31,61 |
| Living while Dying | Filmmaker | 4 | 0 | 4 | 4 | 0 | 0:45:10 | 0:17:46 | 39,34 |
| Living your Dying | Institution | 3 | 2 | 1 | 2 | 1 | 0:56:56 | 0:26:37 | 46,75 |
| Love in Our Own Time | Patient | 5 | 2 | 3 | 5 | 0 | 1:34:00 | 0:47:37 | 50,66 |
| Mortal Lessons | Institution | 2 | 2 | 0 | 2 | 0 | 0:57:02 | 0:19:43 | 34,57 |
| Prison Terminal: The Last Days of Private Jack Hall | Patient | 1 | 0 | 1 | 1 | 0 | 0:44:00 | 0:26:20 | 59,85 |
| Seven Songs for a Long Life | Patient | 6 | 4 | 2 | 6 | 0 | 1:22:40 | 1:07:06 | 81,17 |
| Solace: Wisdom of the Dying Documentary | Institution | 8 | 6 | 2 | 3 | 5 | 1:27:11 | 0:33:07 | 37,99 |
| The End | Patient | 5 | 2 | 3 | 3 | 2 | 1:24:26 | 0:52:53 | 62,63 |
| The Light Inside | Institution | 2 | 1 | 1 | 2 | 0 | 0:24:39 | 0:08:17 | 33,60 |
| The LuLu Sessions | Filmmaker | 1 | 1 | 0 | 1 | 0 | 1:26:34 | 0:47:22 | 54,72 |
| The Perfect Circle | Patient | 2 | 1 | 1 | 2 | 0 | 1:15:39 | 0:48:33 | 64,18 |
| Tuntemattoman edessä | Patient | 1 | 1 | 0 | 1 | 0 | 0:28:10 | 0:26:05 | 92,60 |
| **In total** |  | **135** | **73** | **62** | **107** | **28** | **39:10:37** | **19:52:38** | **50,74** |

Supplemental table 2. The research data quantified at the level of the represented terminally ill patients. Here, each character is listed under their first name, their gender, estimated age race/ethnicity and personal voice time in the narration.

*Age is recorded as provided in the documentary or estimated. The estimated ages are in round numbers; i.e. 40 implies that person is most likely in their 40s.

| **Documentary** | **Patient** | **Gender** | **Age group** | **Ca. age*** | **Race** | **Personal voice time** |
| --- | --- | --- | --- | --- | --- | --- |
| A Good Death | Darryl | Man | Working age | 46 | White | 0:06:33 |
| A Good Death | John | Man | Working age | 53 | White | 0:03:47 |
| A Good Death | Norma | Woman | Elderly | 80 | White | 0:01:37 |
| A Good Death | Sandy | Woman | Working age | 63 | White | 0:05:02 |
| Bearing Witness: Robert Coley-Donohue | Robert | Man | Elderly | 70 | White | 1:05:48 |
| Before We Go | Benoit | Man | Working age | 60 | White | 0:15:40 |
| Before We Go | Lydia | Woman | Working age | 60 | White | 0:23:00 |
| Before We Go | Noel | Man | Elderly | 70 | White | 0:17:08 |
| Being Mortal | Bill | Man | Working age | 46 | White | 0:08:16 |
| Being Mortal | Jeff | Man | Elderly | 80 | White | 0:06:22 |
| Being Mortal | Norma | Woman | Elderly | 80 | White | 0:02:55 |
| Bouton | Johana | Woman | Working age | 30 | White | 1:05:36 |
| Consider the Conversation 2: Stories about Cure, Relief, and Comfort | Al | Man | Working age | 61 | Black | 0:03:08 |
| Consider the Conversation 2: Stories about Cure, Relief, and Comfort | Chuck | Man | Elderly | 92 | White | 0:00:15 |
| Consider the Conversation 2: Stories about Cure, Relief, and Comfort | Deborah | Woman | Working age | 62 | White | 0:00:15 |
| Consider the Conversation 2: Stories about Cure, Relief, and Comfort | Dwight | Man | Working age | 60 | Black | 0:00:45 |
| Consider the Conversation 2: Stories about Cure, Relief, and Comfort | George | Man | Working age | 56 | White | 0:03:43 |
| Consider the Conversation 2: Stories about Cure, Relief, and Comfort | Greg | Man | Working age | 46 | White | 0:00:43 |
| Consider the Conversation 2: Stories about Cure, Relief, and Comfort | Iris | Woman | Working age | 54 | White | 0:00:05 |
| Consider the Conversation 2: Stories about Cure, Relief, and Comfort | Kenneth | Man | Elderly | 75 | White | 0:00:06 |
| Consider the Conversation 2: Stories about Cure, Relief, and Comfort | Laura | Woman | Working age | 52 | White | 0:08:45 |
| Consider the Conversation 2: Stories about Cure, Relief, and Comfort | Paula | Woman | Elderly | 84 | White | 0:00:09 |
| Consider the Conversation: A Documentary on a Taboo Subject | Dee | Woman | Working age | 60 | White | 0:01:17 |
| Consider the Conversation: A Documentary on a Taboo Subject | Martin | Man | Working age | 55 | White | 0:08:54 |
| Consider the Conversation: A Documentary on a Taboo Subject | Pete | Man | Working age | 40 | White | 0:02:55 |
| Dying at Grace | Carmela | Woman | Elderly | 80 | White | 0:10:10 |
| Dying at Grace | Eda | Woman | Working age | 60 | White | 0:38:25 |
| Dying at Grace | Joyce | Woman | Elderly | 80 | White | 0:25:02 |
| Dying at Grace | Lloyd | Man | Working age | 40 | White | 0:25:35 |
| Dying at Grace | Richard | Man | Working age | 60 | White | 0:25:37 |
| En god død | Britt | Woman | Working age | 50 | White | 0:19:36 |
| En god død | Hanne | Woman | Elderly | 80 | White | 0:14:46 |
| En god død | Myrna | Woman | Elderly | 70 | White | 0:18:34 |
| En god død | Unknown | Woman | Elderly | 70 | White | 0:02:10 |
| End Game | Bruce | Man | Elderly | 66 | Asian | 0:00:50 |
| End Game | Kym | Woman | Elderly | 70 | White | 0:01:21 |
| End Game | Mitra | Woman | Working age | 45 | White | 0:11:36 |
| End Game | Pat | Woman | Working age | 50 | Black | 0:04:37 |
| End Game | Thekla | Woman | Working age | 60 | White | 0:02:41 |
| Except for Six | Naomi | Woman | Elderly | 69 | White | 0:00:34 |
| Except for Six | Ron | Man | Elderly | 69 | White | 0:14:28 |
| Except for Six | Rosie | Woman | Elderly | 92 | Black | 0:00:01 |
| Facing Death | Albert | Man | Working age | 53 | White | 0:02:41 |
| Facing Death | Condolena | Woman | Working age | 60 | Black | 0:00:17 |
| Facing Death | Diane | Woman | Working age | 31 | Black | 0:00:26 |
| Facing Death | John | Man | Working age | 55 | White | 0:05:29 |
| Facing Death | Martha | Woman | Elderly | 86 | Black | 0:01:32 |
| Facing Death | Norman | Man | Working age | 60 | Black | 0:01:53 |
| Facing Death | Robert | Man | Working age | 53 | White | 0:00:13 |
| Griefwalker | Kathy | Woman | Working age | 50 | White | 0:03:39 |
| Griefwalker | Sandra | Woman | Working age | 60 | White | 0:08:14 |
| Griefwalker | Sasha | Woman | Child | 6 | White | 0:00:16 |
| Helen's Story | Helen | Woman | Working age | 30 | Indigenous | 0:31:19 |
| Hirschhausen im Hospiz | Alfons | Man | Elderly | 70 | White | 0:01:01 |
| Hirschhausen im Hospiz | Geposki | Woman | Working age | 50 | White | 0:01:43 |
| Hirschhausen im Hospiz | Unknown | Woman | Elderly | 80 | White | 0:00:51 |
| Hirschhausen im Hospiz | Unknown | Woman | Working age | 40 | White | 0:02:26 |
| I Remember When I Die | Bodil | Woman | Elderly | 70 | White | 0:13:20 |
| I Remember When I Die | Finn | Man | Working age | 60 | White | 0:10:56 |
| I Remember When I Die | Lilly | Woman | Elderly | 70 | White | 0:02:23 |
| I Remember When I Die | Ole | Man | Working age | 60 | White | 0:08:47 |
| I Remember When I Die | Susan | Woman | Elderly | 70 | White | 0:00:36 |
| Inside the Human Body: First toLast | Gerald | Man | Elderly | 84 | White | 0:03:23 |
| Island | Alan | Man | Elderly | 80 | White | 0:26:55 |
| Island | Jamie | Man | Working age | 30 | White | 0:16:41 |
| Island | Mary | Woman | Elderly | 80 | White | 0:06:40 |
| Island | Roy | Man | Working age | 60 | White | 0:09:03 |
| Kuoleman kasvot | Jussi | Man | Working age | 50 | White | 0:06:14 |
| Kuoleman kasvot | Mari | Woman | Working age | 50 | White | 0:13:15 |
| Kuoleman kasvot | Raimo | Man | Working age | 60 | White | 0:00:12 |
| Kuoleman kasvot | Tuure | Man | Elderly | 70 | White | 0:01:57 |
| Les Pal·liatives | Antonio | Man | Elderly | 70 | White | 0:05:28 |
| Les Pal·liatives | Francesc | Man | Elderly | 80 | White | 0:04:32 |
| Les Pal·liatives | Maria | Woman | Elderly | 70 | White | 0:03:34 |
| Les Pal·liatives | Marta | Woman | Working age | 19 | White | 0:06:36 |
| Les Pal·liatives | Ramon | Man | Elderly | 80 | White | 0:04:35 |
| Letzte Saison - wenn es Zeit ist zu sterben | Gisela | Woman | Working age | 65 | White | 0:37:56 |
| Letzte Saison - wenn es Zeit ist zu sterben | Roderich | Man | Elderly | 80 | White | 0:19:21 |
| Letzte Saison - wenn es Zeit ist zu sterben | Rubert | Man | Elderly | 87 | White | 0:10:50 |
| Letzte Tage, gute Tage - Palliativ-Versorgung in Deutschland | Gerold | Man | Working age | 48 | White | 0:00:54 |
| Letzte Tage, gute Tage - Palliativ-Versorgung in Deutschland | Matthias | Man | Working age | 60 | White | 0:00:40 |
| Life before Death | Bernard | Man | Working age | 60 | Asian | 0:06:47 |
| Life before Death | Carmen | Woman | Working age | 50 | White | 0:00:04 |
| Life before Death | Don | Man | Working age | 50 | White | 0:02:16 |
| Life before Death | Gomathy | Woman | Working age | 60 | Asian | 0:02:22 |
| Life before Death | Jessica | Woman | Child | 16 | Asian | 0:01:51 |
| Life before Death | Joyce | Woman | Working age | 40 | Black | 0:01:36 |
| Life before Death | Kwai Son | Man | Child | 8 | Black | 0:00:24 |
| Life before Death | Letha | Woman | Working age | 50 | Asian | 0:01:18 |
| Life before Death | Lorraine | Woman | Elderly | 70 | White | 0:01:10 |
| Life before Death | Maria | Woman | Working age | 40 | White | 0:01:57 |
| Life before Death | Mildred | Woman | Working age | 50 | Black | 0:01:19 |
| Life before Death | Suseela | Woman | Elderly | 80 | Asian | 0:03:33 |
| Life before Death | Teena | Woman | Working age | 40 | Black | 0:00:18 |
| Life before Death | Willy | Man | Working age | 40 | Asian | 0:00:42 |
| Living while Dying | AvYitz | Man | Working age | 50 | White | 0:05:34 |
| Living while Dying | Azul | Man | Elderly | 70 | White | 0:02:34 |
| Living while Dying | Clair | Man | Elderly | 80 | White | 0:04:22 |
| Living while Dying | Don | Man | Working age | 50 | White | 0:05:16 |
| Living your Dying | Fay | Woman | Working age | 50 | White | 0:08:12 |
| Living your Dying | Mike | Man | Elderly | 70 | White | 0:07:37 |
| Living your Dying | Ululani | Woman | Working age | 50 | Indigenous | 0:10:48 |
| Love in Our Own Time | Douglas | Man | Elderly | 80 | White | 0:16:02 |
| Love in Our Own Time | John | Man | Working age | 50 | White | 0:18:52 |
| Love in Our Own Time | Jutta | Woman | Elderly | 70 | White | 0:06:21 |
| Love in Our Own Time | Noelene | Woman | Elderly | 70 | White | 0:04:37 |
| Love in Our Own Time | Walter | Man | Elderly | 80 | White | 0:01:45 |
| Mortal Lessons | Carole | Woman | Working age | 62 | White | 0:05:24 |
| Mortal Lessons | Cindy | Woman | Working age | 60 | White | 0:14:19 |
| Prison Terminal: The Last Days of Private Jack Hall | Jack | Man | Elderly | 82 | White | 0:26:20 |
| Seven Songs for a Long Life | Alicia | Woman | Elderly | 70 | White | 0:08:29 |
| Seven Songs for a Long Life | Dorene | Woman | Working age | 40 | White | 0:06:40 |
| Seven Songs for a Long Life | Iain | Man | Working age | 40 | White | 0:10:58 |
| Seven Songs for a Long Life | Julie | Woman | Working age | 30 | White | 0:14:39 |
| Seven Songs for a Long Life | Nicola | Woman | Working age | 40 | White | 0:17:39 |
| Seven Songs for a Long Life | Tosh | Man | Elderly | 70 | White | 0:08:41 |
| Solace: Wisdom of the Dying Documentary | Anne | Woman | Elderly | 80 | White | 0:04:38 |
| Solace: Wisdom of the Dying Documentary | Chizuko | Woman | Working age | 60 | Asian | 0:04:54 |
| Solace: Wisdom of the Dying Documentary | Chris | Woman | Working age | 50 | Black | 0:06:02 |
| Solace: Wisdom of the Dying Documentary | Diego | Man | Working age | 60 | Asian | 0:04:03 |
| Solace: Wisdom of the Dying Documentary | Gertrude | Woman | Elderly | 80 | Latinx | 0:01:29 |
| Solace: Wisdom of the Dying Documentary | Kathy | Woman | Working age | 50 | White | 0:06:13 |
| Solace: Wisdom of the Dying Documentary | Mary | Woman | Working age | 50 | White | 0:03:10 |
| Solace: Wisdom of the Dying Documentary | Ricardo | Man | Elderly | 80 | Latinx | 0:02:38 |
| The End | Phil | Man | Elderly | 70 | Black | 0:12:40 |
| The End | Robert | Man | Working age | 50 | White | 0:10:38 |
| The End | Rosalie | Woman | Working age | 50 | White | 0:11:10 |
| The End | Stan | Man | Elderly | 70 | White | 0:12:43 |
| The End | Susie | Woman | Working age | 19 | Latinx | 0:05:42 |
| The Light Inside | Ethan | Man | Child | 6 | White | 0:02:21 |
| The Light Inside | Patti | Woman | Working age | 50 | White | 0:05:56 |
| The LuLu Sessions | Lulu | Woman | Working age | 40 | White | 0:47:22 |
| The Perfect Circle | Ivano | Man | Elderly | 70 | White | 0:29:07 |
| The Perfect Circle | Maris | Woman | Working age | 60 | White | 0:19:26 |
| Tuntemattoman edessä | Jaana | Woman | Working age | 30 | White | 0:26:05 |
